# Disturbance of consciousness caused by dyclonine hydrochloride mucilage: a case report

**DOI:** 10.1186/s12871-024-02407-x

**Published:** 2024-01-22

**Authors:** Li Wang, Qi-Hui Wen, Li-Juan Wen, Jia-Min Qin, Chun-Mei Ren, Li-Ming Wen

**Affiliations:** 1 Department of Gastroenterology, Sichuan Mianyang 404 Hospital, Mianyang, Sichuan 621000 China; 2 Department of infectious diseases, Sichuan Mianyang 404 Hospital, Mianyang, Sichuan 621000 China

**Keywords:** Adverse effect, Disturbance of consciousness, Dyclonine hydrochloride mucilage

## Abstract

**Background:**

Dyclonine hydrochloride mucilage is a topical anaesthetic formulated for mucosal surfaces. It is employed frequently for topical anaesthesia of the pharynx prior to endoscopic examinations such as electronic gastroscopy, and few adverse reactions have been reported. This article describes a patient who experienced a transient but severe disturbance of consciousness following oral dyclonine hydrochloride mucilage administration.

**Case presentation:**

A 75-year-old female presenting with gastrointestinal bleeding was examined by electronic gastroscopy. Six minutes after oral dyclonine hydrochloride mucilage administration, the patient entered a comatose-like state accompanied by loss of limb muscle tone and profuse perspiration. This response was not accompanied by changes in cardiac rhythm, blood pressure, or respiration rate, suggesting an effect on higher brain centres. After ten minutes, the patient’s symptoms were alleviated.

**Conclusion:**

We suggest that sites of dyclonine hydrochloride mucilage use be equipped with appropriate rescue devices for these rare events.

## Background

Dyclonine (4-butoxy–piperidinyl phenylacetone hydrochloride) has been used clinically since 1956 as a local anaesthetic due to low toxicity, rapid onset, and a good safety profile [1]. It is a ketone-based compound structurally distinct from many other common local anaesthetics such as lidocaine and procaine, and therefore it can be safely administered to patients with procaine allergies [2, 3]. The dyclonine hydrochloride mucilage formulation is extensively utilized for topical anaesthesia of the throat prior to endoscopic examinations such as electronic gastroscopy. While reactions such as nausea and vomiting have been reported, disturbance of consciousness is a very rare adverse effect.

## Case presentation

**Fig. 1:**
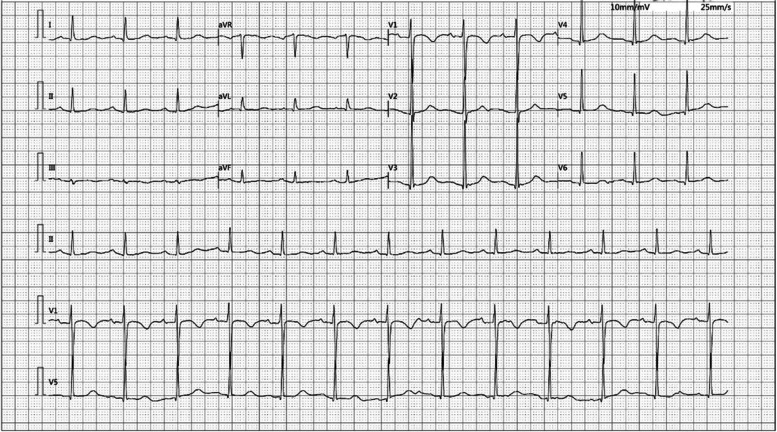
Electroencephalograph showing normal sinus rhythm during the reaction to oral dyclonine mucilage

A 75-year-old female (height 1.56 m, weight 44 kg) with a history of hepatitis B infection was admitted to Sichuan Mianyang 404 Hospital (Sichuan Province, China) in October 2022 due to a one-week period of black stool. Upon physical examination, the patient was fully alert and vital signs were within normal ranges (body temperature, 36.7 °C; heart rate, 91 bpm; respiration rate, 20 per min; blood pressure, 143/75 mmHg). The diagnosis at admission was gastrointestinal haemorrhage, decompensated cirrhosis following hepatitis B infection, and hypersplenism. On October 24, 2022, electronic gastroscopy was performed, and dyclonine hydrochloride mucilage (Yangtze River Pharmaceutical Group, Jiangsu, China; lot number: 22,121,511, specification: 0.1 g in 10 mL) was administered orally at 17:00. At 17:06, the patient experienced abrupt cognitive impairment, limb weakness, and profuse sweating, but there were no marked changes in heart rate (90 bpm), respiration rate (19 times per min), or blood pressure (148/76 mmHg). Physical examination during the event revealed coma and loss of muscle strength in the limbs but no obvious pathological symptoms. Further, electrocardiography revealed no abnormalities in cardiac waveforms (Fig. 1), while blood sugar was high-normal (10.5 mmol/L).

**Fig. 2:**
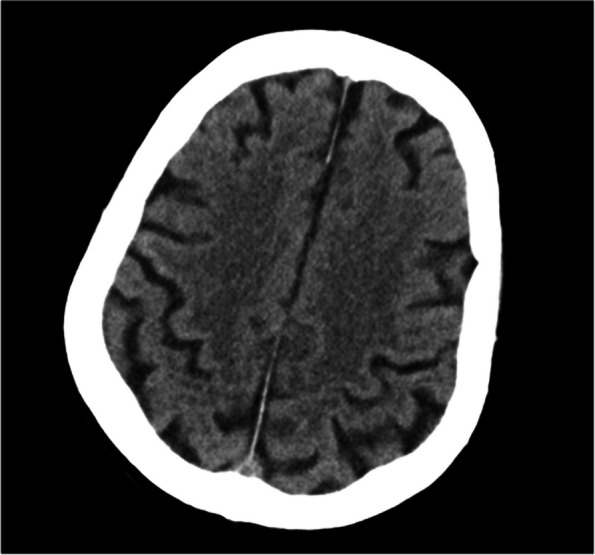
Head computed tomography scan showing bilateral lacunar cerebral infarction in the basal ganglia

Inhaled oxygen was initiated immediately and a venous channel was established for fluid replacement. Altered consciousness started to improve at 17:10, and the patient was responsive to verbal communication thereafter. Cranial computed tomography scan revealed bilateral lacunar cerebral infarction in the basal ganglia (Fig. 2); therefore we sought immediate consultation from the Neurology Department. However, no special treatment was deemed necessary given the stable vital signs, including unchanged haemoglobin (82 g/L) and absence of specific symptoms and indications of neurological deficits. At 17:15, muscle strength in the extremities was grade 3. At 18:00, both mental state and muscle strength had fully recovered. The patient’s family also reported that on September 7, 2021, prior to another gastroscopy examination, oral dyclonine hydrochloride mucilage induced transient dizziness and loss of consciousness, followed by recovery without medical attention. The patient underwent a second gastroscopy at our institution under general anaesthesia on October 26, 2022, without the use of dyclonine hydrochloride mucilage, the disorder of consciousness did not reappear.

## Discussion

Before anaesthesia, the case patient was alert and healthy but experienced rapid transient loss of consciousness and limb strength roughly six minutes after ingesting dyclonine hydrochloride mucilage. The patient received no other medications, so this temporal correlation indicates that dyclonine mucilage caused transient mental confusion, a coma-like state, and loss of limb strength.

Dyclonine binds reversibly to activated (open) sodium channels on the neuronal and axonal membrane, thereby blocking the transmission of action potentials encoding pain and other sensory signals controlling pharyngeal reflexes [4]. Common adverse effects of dyclonine include nausea, dizziness, and vomiting, while occasional side-effects include mild headache, anxiety, cold or hot sensations, and numbness. A previous report found that intravenous injection of 200–500 mg dyclonine had no apparent adverse effects [5]. The dyclonine hydrochloride mucilage formulation also contains sodium carboxymethyl cellulose to increase liquid viscosity and facilitate even spread across the mucosal surface. In this form, the mucosal anaesthesia effect is both rapid in onset (2–10 min) and long-lasting (2–4 h) but with negligible central effects. Dyclonine hydrochloride mucilage can reduce throat spasm, eliminate mucus bubbles in the oropharyngeal cavity, and produce a defoaming effect in the gastric cavity, thereby improving the field of vision during electronic gastroscopy [6]. Further, numerous studies have confirmed better efficacy and safety compared to lidocaine and tetracaine for gastrointestinal endoscopy and tracheal intubation [2, 7].

The patient developed symptoms after taking dyclonine hydrochloride mucilage twice. The first time, the symptoms were temporary dizziness and loss of consciousness, which quickly alleviated. The second time, the symptoms were severe, necessitating a consultation of relevant literature. There are currently few reports on severe adverse reactions resulting from the use of dyclonine hydrochloride mucilage. Gu and colleagues reported a case of respiratory failure five minutes after local anaesthesia with dyclonine hydrochloride mucilage for electronic laryngoscopy [8], while Gao and Huang reported a case of dizziness, blurred vision, tinnitus, fatigue, nausea and vomiting with foamy mucus, and weakness of limbs after administration of dyclonine hydrochloride mucilage by gastroscope for radical total gastrectomy [9]. In this latter case, however, no convulsions, dyspnoea, or mental confusion were reported, the male case patient was able to respond to verbal statements throughout, and symptoms were alleviated following treatment with dexamethasone and other antiallergic medications [9]. Hu and colleagues [10] reported a case of mania while Jia and co-workers [11] reported a protracted QT interval and T wave inversion following dyclonine hydrochloride mucilage administration. Finally, Cheng and Huang reported several instances of anaphylaxis following dyclonine hydrochloride mucilage administration [12, 13]. The adverse reactions of dyclonine hydrochloride mucilage are generally milder, such as dizziness, nausea, vomiting, limb weakness, and so on, severe allergic reactions are rare, and anti-allergic drugs should be used. This patient did not use anti-allergic drugs, and the symptoms alleviated quickly, so it could not be explained by allergic reaction.

All patients in our hospital who received dyclonine hydrochloride mucilage (0.1 g) before gastroscopy had no adverse reactions, indicating that this dose is safe. The patient received dyclonine hydrochloride mucilage before gastroscopy without using other anesthetic drugs, so the symptoms were not caused by a drug cross-reaction. Cerebrovascular and cardiogenic diseases, endocrine and metabolic disorders, exogenous poisoning, and acute infectious diseases can all result in disturbances of consciousness. However, In this case, none of these disorders is a likely explanation. It is worth noting that this patient had liver disease and a low body weight (44 kg), which raises the question whether the above reaction after the administration of dyclonine hydrochloride mucilage was caused by an acute toxic reaction. The Australia Therapeutic Goods Administration has issued a safety bulletin warning of systemic toxicity from local anesthetics. The symptoms include mental state changes (i.e., anxiety), muscle tremor, loss of consciousness, and mild hemiplegia. The risk factors for local anesthetics systemic toxicity include liver insufficiency. The reported incidence of major local anesthetic systemic toxicity events associated with regional anesthesia is very low [14].

In this case, the patient experienced discomfort after the initial use and continued to exhibit symptoms upon subsequent exposure. The patient’s symptoms appeared 6 min after taking the drug, found alleviate after 4 min, and felt significantly better after 1 h, which is consistent with the time of drug metabolism. The patient’s second symptom was more serious compared to the initial one. It is considered that after the first drug stimulation, the patient became more sensitive to the drug, so the symptoms were aggravated. For patient with profuse sweat, it is considered to be a functional sympathetic excitement caused by anxiety. This patient is an elderly woman, often anxious and fearful of gastroscopy, so she experienced profuse sweat. The patient’s symptoms were consistent with systemic toxicity caused by dyclonine hydrochloride mucilage, and the duration of these symptoms was consistent with the drug’s metabolism time. Therefore, the patient’s symptoms were considered to be caused by acute toxic reaction of dyclonine hydrochloride mucilage.

## Conclusion

This report indicates that topical application of dyclonine hydrochloride mucilage can induce mental confusion and transient loss of consciousness. The patient’s vital signs and level of consciousness should be closely and continuously monitored after each local anesthesia. Therefore, clinical sites where dyclonine mucilage is administered should be routinely stocked with rescue equipment and medications.

## Data Availability

Not applicable.
